# Professors Mansfeld, Schenk, and Meyer, on the Antiquity and History of Gastrotomy and Hysterotomy, with Successful Cases of Cæsarean Operation

**Published:** 1826-04

**Authors:** 


1. *Ueber das Alter des Bauch und Gebarmutterschnitts an Lebenden.* Von Dr. Mansfeld, zu Braunschweig, 1824.

*On the Antiquity of Gastrotomy and Hysterotomy on the Living.* By Dr. Mansfeld of Brunswick, 1824.

2. *Geschicte einer glucklichen Entbindung durch den Kaiserschnitt.* Von Dr. J. H. Schenk, zu Siegen. Frankfurt, A. M. 1826.

*History of a successful Delivery by the Cæsarian Operation.* By Dr. J. H. Schenk of Siegen. Frankfort on the Mayne, 1826, pp. 230.

3. *Geschicte einer durch den Kaiserschnitt glucklich biendigten Entbindung.* Von Nicolaus Meyer, Doctor der Medicin und Chirurgie, &c. in (Preussisch) Minden.

*History of a Delivery by the Cæsarian Operation which terminated successfully.* By Dr. Meyer of (Prussian) Minden, pp. 74.

The earliest hitherto admitted record of what is now called the Cæsarian section on a living woman, is to be found in the Observationes Chirurgicæ, published at Venice in 1491, by Nicolai de Falconiis. Stein, Simon Osiander Loder, and other obstetric writers have omitted to notice this case, and have referred to the operation performed by Jacob Niefer, a Swiss peasant on his own wife, in the beginning of the sixteenth century, as the first. According to Kurt Sprengel,* we are not to consider the operation

* Geschicte der Chirurgie, 1 Theil. Halle, 1803.

to have been performed on the living subject earlier than 1610, when the experiment was made by Jeremias Trautmaun. Some have altogether denied that the operation was known to the ancients, among whom are DELEURYE, LEVRET, MAURICEAU, and MENDEL, whilst others, as for example JOSEPH PLENK, DIONIS and GARDIEN, content themselves with quoting the passage in the 34th book of Pliny's Natural History, in which he mentions that Scipio Africanus and Manlius were born by cutting into the womb, but it is evident that this was done after the death of the woman. That the operation was performed long before the period admitted by Sprengel is very evident. ROUSSET, who was physician to Catharine de Medicis, and a cotemporary of Ambrose Paré, collected together the history of many successful cases of the cæsarian operation on the living, and published them at Paris in 1581.* Rousset's book was translated into Latin, by CASPAR BAUHIN† of Basil, in 1591, who added to the number of cases, and confirmed those already collated by Rousset. The Latin translation was more extensively circulated than the original work, and the consequence was, that the subject excited considerable attention, and the operation, notwithstanding the stern opposition of Paré, whose opinion at that time had great weight, continued to be performed throughout France and Germany. DIONIS and SACOMBE have been, in France, the greatest opponents to the operation, and Sacombe, through the notorious *Lucine Française*, a publication which undertook to expose all the unfortunate cases which occurred in the practice of the surgeons of Paris, stigmatized Rousset as an imposter, and those who credited him, as madmen. He went farther, and ascribed the worst of all possible motives to Rousset for recommending the operation, "François Rousset," said Sacombe, "en propageant une operation toujours mortelle eut le double motif du fanatisme et de l'ambition en faisant egorger les femmes des protestans, pour plaire a Medicis qui en reconnaissance des ses bons offices le fit médécin de sa cour."‡ In this country the operation has been defended by some, and is warmly opposed by others abstracted from all qualifications and conditions which indicate and contra-indicate its performance. It must, however, be at present admitted, that such an operation is occasionally necessary, and it is a matter of regret, that the history of modern surgery, leaves us nothing in this respect to boast of over the practice of our predecessors.

This then is the generally received history of the origin and progress of the operation on the living, and we shall in the course of this article, show the arguments adduced by Dr. Mansfeld, to prove its still higher antiquity; take a glance at the methods recommended for its performance; and lastly give such results of the operation as we have been able to collect.

The laws of the ancient Egyptians, Jews, Grecians and Romans, not only sanctioned, but prescribed the opening of the uterus through the abdomen in all cases of advanced pregnancy after the death of the female so pregnant; but that the same sanction was allowed to the performance of the operation during life has been hitherto denied. A position which Dr. Mansfeld by his

* Traité nouveau de l'Hysterotomakie ou Enfontany Cæsarienne, Paris.

† A very curious and amusing compilation, it is entitled Fœtus vivi ex *matre viva* sine alterius vitæ periculo cæsura a Francisco Rousseto conscripta, Casparo Bauhino latiné reddita, variis historiis aucta et confirmata, Basil, 1591, 8vo. fol. 117. A great part of the substance of this book is to be found in the Commentarii Gynaciorum. Tom. ii. 1586.

‡ Elemens de la Science des Accouchemens, par Sacombe, p. 283. Lucine Française, No. ii.

researches in Hebrew literature has undertaken to controvert; with what success the following passages will shew. It is very natural that the first reference should be made to the Thalmud, as it embodies, according to the admission of all Hebrew scholars, the learning, and for the most part, the laws of the ancient Jews. In it are to be found several long chapters on the subject of hereditary rights, in which these cases are particularly considered where delivery was effected by gastrotomy during life. Yet earlier even than the Thalmud was the Mischnajoth compiled, and it may be safely considered as an authentic record of many of the customs and ceremonies of the Jewish nation; it is admitted to have been the joint labour of many learned Rabbis, who lived about the year 140. In the eighth chapter of the Mischnajoth on the rights of the first born, the birth by gastrotomy, or as the Hebrew words express it ירצא רופן is distinctly spoken of and discussed: the passage literally translated is as follows, "In a twin birth, neither the first child, which by the section of the belly is brought into the world, nor the one coming after can attain the rights of heirship or the priestly office." The Mischnajoth contained many commentaries on certain passages of the Thalmud, and passages in the former were also commented upon by later writers. Bartenora,* a learned Hebrew, who wrote much on the literature of his country, gives such an explanation of the first part of the passage above cited as puts the purport of it beyond all doubt. "Should a woman" says he, "be pregnant with twins, and the first be delivered through an opening made in the *side of the belly, such a woman can never again, according to the opinion of Maimonides, bear children.*" Schaliger, in his Epistles, and Blumenbach† in his Introduction to the History of Medicine, make mention of the last named author; he was a man of great learning, and a public teacher of medicine and mathematics in the University of Corduba in Spain, so early as the year 1170. "It is possible" says Maimonides, "that a woman may be delivered of twins, the one through an opening made in the side, and the other by the natural way; but the opinion of those is without all reason, who hold that such a woman can again become pregnant and be delivered." If these passages should leave any doubt of the operation having been performed on the living, in the minds of the sceptical, the following, from a very old Hebrew work called Nidda, written in the fourth century, must remove them. The Nidda was written by Rabbi Asche, a teacher at Sora, as an Appendix to the Thalmud, and chiefly on the religious rites of women; the passage is as follows: "After gastrotomy (for we can find no better synonyme for the Hebrew words) it is not necessary for the women to observe the days of purification." Nothing can be more plain than that the Cæsarian operation was performed on the Jewish women during life, and from the opinions expressed by the commentators of the Thalmud, and the author of the Nidda, there is good ground for presuming that it must occasionally have been attended with success. Several other passages equally forcible are brought forward by Dr. Mansfeld, to bear upon this point, which our limits will not allow us to insert; he has shewn an intimate acquaintance with Hebrew literature, and has produced an interesting addition to the history of obstetric surgery.

The mode of performing the Cæsarian operation, properly so called, has

---

* Obadja Bartenora, was a native of Bertinoro in Italy; he lived in the latter part of the fifteenth, and the beginning of the sixteenth century.—*See Yung's Alphabetischi Liste der gelehrten Juden Leipseig*, 1817.

† Fried. Blumenbachii Introductio in Historiam Medicinæ Literariam, p. 94.

been frequently varied, according to the peculiar doctrines and ingenuity of the operators, but the methods which have been most practised are four: 1st, The lateral incision; 2d, The incision in the linea alba; 3d, The transverse; and 4thly, The diagonal incision.

Of these, the *first*, or the *lateral incision*, is the most ancient, as appears from the authorities last quoted. Rousset, however, is entitled to whatever merit or demerit may attach itself to the publication of this mode of operating, and, according to him, the incision to be made in the abdominal parietes, must be commenced immediately under the umbilicus, and continued downwards and outwards, parallel to the outer edge of the rectus, until the lower part of the incision is at the distance of three fingers breadth from the linea alba. Levret, in 1759, and Stein still later, described more accurately the manner in which the lateral operation was performed. The incision is to be made, according to them, on that side in which the projection of the fundus uteri can be most distinctly felt, the incision through the parietes to be commenced in a horizontal line with the umbilicus midway between a line drawn from the union of the last true rib with its cartilage to the anterior superior spinous process of the ilium and the linea alba, so that the incision was made at the distance of about two fingers' breadth from the linea alba. Stein* recommended that the integuments should be first divided the whole length required, and afterwards, that the muscles and peritoneum should be gradually divided by introducing a bistoury on the finger, and thus, to enlarge the opening to the necessary extent upwards and downwards to the same length as the external wound, which was from six to seven inches long. Millott recommended the incision to be made on either side of the abdomen, according as the uterus might be more distinctly felt, but that it should extend from the cartilaginous edge of the last false rib to within one inch of the pelvis, assigning as a reason for so doing, the greater ease allowed to the operator of pushing back the prolapsed intestines, appearing to have forgotten that such an inconvenience must be occasioned by the very means which he recommended for its removal.† Thus the lateral operation was considered perfected by those who advocated it, and remained, on the Continent, during the greater part of the 18th century, the most prevalent mode of operating.

*Secondly*—Of the incision in the *linea alba*. It was not until toward the close of the eighteenth century, namely, between 1770 and 1780, that some French and German surgeons began to practise the operation in the linea alba, although Mauriceau had, so early as 1721, recommended it, and had employed a very strong argument to support his proposition. "Men generally recommend" says Mauriceau, "the opening to be made in the side, but it is much better to make it between the recti muscles, for there are only muscles and integuments to divide." This advice appears to have been lost sight of for many years, and when Guenin and L. Platner published their observations on the advantages of the linea alba as a place for operation; they were regarded for some time as the discoverers.

Platner accurately described such a mode of operating. "Incidantur juxta lineum album plagâ majore quæ ab umbilico ad ossa pubis ferè descendit, &c." but whether Platner ever performed such an operation is not so certain. Guenin, however, did, and his history of two cases of Cæsarian

---

* Stein's Verfohren in Abhandlung, v. d. Kaiserg. in Klinen Schriften Morburg, 1798.

† Millot, Observations sur l'Operation Cæsarienne avec la Description d'une nouvelle Methode.—*No.* vii. *Tome*, i. *Annales de Chirurgie.*

operation in the linea alba made their appearance in 1750.* Guenin was the first who performed the operation in France. DELEURYE† many years after published some observations on the linea alba operation, in which he refused to admit that Guenin had before performed it, and named Varoquier, a surgeon at Lisle, as the discoverer. Deleurye enforced the superiority of this place for the operation, on account of the less danger of hæmorrhage, the greater facility of drawing the wound together, the less risk of injuring the intestines, and the greater readiness allowed to the escape of the discharge, and since that time, the operation has been generally adopted. The operation itself is very simple; a perpendicular incision is made through the integuments over the linea alba, the parietes of the abdomen and uterus are then cut through, and that part of the child laid hold of which may happen to be uppermost. DELEURYE and BAUDELOCQUE have differed a little about the length of the incision which is necessary, and OSIANDER proposed to divide the lower two thirds of the uterus only, instead of the upper as before practised. The operation as it is at present performed, consists of making an incision through the linea alba, having first secured the uterus firmly in its position, to prevent the intestines slipping between it and the fore part of the abdominal covering, from the umbilicus to within an inch of the symphysis pubis; the uterus is next cut into nearly to the same extent, and the child gently laid hold of and extracted. This operation was performed in Germany for the first time by Henkel in 1769.

The *third* mode of operating, or the *transverse incision* was publicly proposed by LAUVEIJAT,‡ in 1788, and he acknowledges, that it was shown him by a country surgeon who had successfully operated in this manner. Lauveijat proposed that the uterus should be opened transversely, by making an incision on either side of the abdomen between the rectus muscle and the spine higher or lower in the abdomen as the height of the uterus might make necessary. Lauveijat performed the operation twice in this manner with success, and Mr. Wood, of Manchester, afterwards did a similar operation, an account of which is in the sixth volume of the Medical and Physical Journal, this place was chosen because the nates of the child could be distinctly felt, and it was quite certain that no intestines intervened between the uterus and the abdominal coverings. With the exception of one case more published in Coutouly's Journal de Medecine of 1809, we are not aware that this operation has been further performed.

The *fourth* and last method of operating is that by the *diagonal incision* proposed by STEIN,§ formerly Professor of Midwifery at Morburg, at present in Bonn. It was made in the following manner; the incision was commenced at the bottom of the last false rib of the one side, to the body of the pubis on the other, so that the line thus described intersected the linea alba at its middle.

These are the four principal modes of performing the operation, which have at different times been followed: the linea alba operation, is, however, the one which now prevails on the Continent and in this country, and indeed, we have not heard for many years of the operation having been performed in any other manner, with the exception of two cases after Stein's method, the last of them was published in Mende's Midwifery Journal, during the preceding year. Of the success of the operation, it would be gratifying to be

* Histoire des deux Operations Cæsariennes.—*Paris,* 1750

† Deleurye Observations sur l'Operation Cæsarienne a la ligne blanche, &c.—*Paris,* 1779.

‡ Nouvelle Methode de Pratiquer l'Operation Cæsarienne.—*Paris,* 1788.

§ Stein's Geburtshulfe Abtrondlung Morburg, 1803. 1 Heft. S. 125.

able to say more than the history of surgery will allow us to do. The success formerly was much greater than it has been of late years, a circumstance at which we cannot help feeling surprized when the disadvantage of the situation, the risk of dividing the epigastric artery, and the mechanical unskilfulness of the operators are taken into consideration. KLEIN, who was a very industrious physician and a careful compiler, found that of *eighty-two* Cæsarian operations performed between 1500 and 1769, the period in which the lateral operation prevailed, only *six* proved fatal, or one case in every thirteen and a third. In this country, the success has been very little, and if we except Mr. Barlow's* case of Chorley, we are not acquainted with one which terminated in the preservation of the mother, although the operation has been performed eighteen times.

Dr. Kellie, who published the result of his inquiries into the mortality of this operation, in vol v. of the Edinburgh Medical and Surgical Journal, found that out of 231 cases scattered about in the Records of Surgery, 139 were said to have terminated successfully. The operation has, during the year 1825, been performed in Germany *thrice* with success; once by Dr. SCHENK, a condensed history of which cure is subjoined; once by Professor GRAEFE, a surgeon of great enterprize and talent, at Berlin; and, lastly, by MENDE the successor of OSIANDER and professor of midwifery at Gottingen. The two last cases are not yet published; they will be inserted in the next number of this Journal. Within the same period, the histories of six unfortunate cases have made their appearance, three in Siebold's *Journal fur Geburtshulfe, &c.* and three in Mende's Obstetric Journal, which have a very favourable quotient on the successful side. If we take the number of unfortunate cases that are published, and allow half that number for the cases not published, and then compare these with the successful cases which we may be certain always make their appearance, we shall find that the proportion of the fortunate to the unfortunate will be about one in ten. Such is the result of a careful examination of the published documents up to the present time, and of many extended enquiries which we have instituted on this subject.

The cases which occurred in the practice of Drs. Schenk and Meyer are instructive in several respects; we shall give an outline of each, and first of

*Dr. Schenk's Case.* On the first of July, 1823, Dr. S. was called to a woman in labour; she was about 38 years of age and already the mother of *six* children with each of which she had easy labours. Her last confinement happened in the spring of 1819. During the progress of the present pregnancy the patient had, with a very trifling exception, enjoyed good health. The labour, according to the account of the midwife, commenced on the night of the 29th of June, the pains were slight, the os uteri dilated very slowly, so that the membranes did not break until the following night. The pains became more quick and strong, but the progress of the labour did not keep pace with their violence and frequency, and it was observed that the head of the child did not descend in the pelvis. In the afternoon of the same day the patient remained quiet and the os uteri again gradually contracted. On the arrival of Dr. S., about forty hours after the commencement of the labour, he found the patient free from fever and not much exhausted; she could readily raise herself in bed, when desired, and, without assistance, walk to and fro in the chamber The history of the former accouchemens very much puzzled Dr. Schenk to account for the tardiness and

* Medical Reports and Researches, 1798.

difficulty of the present; but when he tried to pass his hand into the pelvis to ascertain the exact state of the parts, to his very great astonishment, he found that the pelvis was altogether deformed and that the ischia were so pressed together that it was with the greatest difficulty two fingers could be introduced between them. The orifice of the uterus was dilated about the size of a shilling and so high up that it could scarcely be reached. The abdomen, externally, was almost pendulous and projected very much over the pubis. On more minute enquiry into the patient's history, it was found that after her last confinement she was attacked by rheumatism in the hips and loins; the pain attending which was so severe and so long-continued that she lay for several months a cripple in bed. From this complaint she was cured by taking large doses of train oil (the *oleum jecoris aselli* was used in this case*) and was under the management of Dr. Schenk, the uncle of the present practitioner. She so far recovered that she was able to amuse herself a little in the garden in the summer, but always walked crooked and was unable to raise herself upright. A relapse followed, attended with a softening and distortion of the bones, or *osteomalacic*,† as it is termed by the Germans, so that she was obliged to lie a full year in bed on her left side, during which time her back became very much curved. Dr. S. says that, when he was called to the patient on this occasion he found that the spine was very much curved forwards so that the head and chest were almost pressed together; the ribs on the left side were quite flat, and on the right side projecting just as much. The os ilium of the left side was very much pressed inwards. The rami of the ischii and of the pubes were so pressed together that scarcely an inch intervened. The body of the pubis was much pressed inwards and formed with the ramus an acute angle, by which the oblique diameter of the upper and lower orifice of the pelvis was much diminished. The head of the child was, on the preceding evening, fixed in the upper aperture of the pelvis and the os uteri almost closed. After a consultation with most of the respectable practitioners of the neighbourhood, it was determined to perform the Cæsarian operation for the delivery of the child

*The operation* was performed on the 2d of July, at 10 in the morning, in the following manner: The incision was commenced in the linea alba about an inch beneath the umbilicus, and carried downwards in the linea alba, nearly to the symphysis pubis. The wound was between six and seven inches long, and the integuments, as soon as they were divided, separated to a considerable distance. By the second incision, which was made to perfect the division of the abdominal parietes, the uterus was cut into; the vessels which were divided immediately contracted within the structure of the uterus, so that the bleeding was quite insignificant. The uterus was divided to the length of the external wound, by introducing the two first fingers of the left hand, into the small opening made in the upper part, and carrying on the bistoury between them, toward the pubis. Pott's fistula knife was used by Dr. Schenk. No artery spouted, and the blood lost only amounted to a few ounces. The membranes were now cut through and the child exposed. In the upper part of the incision hung a portion of the placenta, which was divided about half an inch deep by the first cut into the uterus; no bleeding of consequence, as before remarked, followed this accident. The child was taken hold of by the right hand and extracted with a gentle rotatory motion. The child, a girl of moderate size, gave, at first, very weak signs of life, the cord was tied as quickly as possible, and

---

* Described in the December Number of Hufeland's Journal of 1822.

divided. Immediately after the child was removed, the uterus suddenly contracted, and the placenta was, in the same moment, partially separated, and inclosed; at the same time, a small quantity of blood was poured out from the surfaces of the wounded uterus, and from the place at which the placenta adhered to the uterus, guided by the cord, the operator carried his finger into the uterus, separated that part of the placenta that remained adherent and speedily removed it. Another small discharge of blood followed its removal. The intestines, which had projected at the sides of the wound in the abdomen were cleansed from the blood with which they became covered during the operation, and were with a little difficulty next returned. The edges of the wound were brought together and six sutures were inserted, about the distance of ¾ of an inch, one from the other; straps of adhesive plaster were also employed, and over them a compress of linen and a bandage were laid. The lower part of the wound was only lightly covered to allow of the escape of the fluid from the uterus and abdomen. The four-headed bandage, which had been previously placed under the patient, was brought over the abdomen to give it the necessary degree of support. The operation itself, and the extraction of the child, occupied three minutes; the bandages were applied, the patient dressed and in bed in ten minutes from the commencement of the operation.

In the first hour afterwards the patient complained of pain in the loins and belly; nauseated a little but did not vomit. The pulse was small but regular; a small quantity of an opiate mixture was given and the patient was allowed a little coffee and a cup of soup. About seven in the evening a few slight after-pains returned with nausea, but passed off without causing any mischief; the patient became covered with a gentle moisture and the circulation and respiration went on tranquilly. The lochia were discharged by the vagina and the lower part of the wound, and towards night the patient was in such a favourable state that it was not considered necessary to administer an opiate. The patient slept well during the night and was only awoke once by the crying of the child. The progress of the case was most favourable; a few unpleasant symptoms occurred, such as a slight cough and colicky pains, which were removed by the proper measures.

On the morning of the 6th of July, ninety hours after the operation, the bandages were, for the first time, removed; no part of the wound could be said to be perfectly united, but of six inches, the original length of the incision, four appeared to be adherent. The edges of the wound were a little reddened and hardened. On the 8th of July, the sutures were loosened. By attentively feeling the part it was discovered that the uterus was adhering to the inner surface of the wound, and at its lower part the bladder could also be distinctly felt to adhere. The case continued to proceed favourably; on the 13th of August she sat up a little for the first time.

On the 22d of August the wound was quite cicatrized with the exception of a little point in the middle about a line in length, but at the end of the month the cure was complete. The cicatrix measured three inches, only one half of the original length of the wound, and the patient was able, without any difficulty, to return to her household affairs. On the 12th of October, three months after the operation, the menses returned, for the first time, and the discharge, instead of being less, was even more, than the ordinary quantity. What was a very curious fact, the discharge not only escaped by the vagina, but also *oozed out* in considerable quantity from the middle and broad part of the *newly formed cicatrix* in the abdomen, at that part at which I had before discovered the uterus to be adherent. Of this fact Dr. Schenk was an eye-witness, and, as far as we know, it has never been before noticed. He says, further, that, through the thin coverings of the part, he could distinctly feel the menstrual congestion of the uterus. In the succeeding menstruation the same phenomenon was observed, the fluid each time diminished in quantity, and, after three months, entirely ceased.

In May, 1824, ten months after the operation, the cicatrix was carefully examined, and it was found that the uterus was quite separated from its former place of adhesion, and that, when the fingers were delicately passed over the parts, the motions of the intestines could be felt beneath. The woman, up to the present time, continues to enjoy good health. This is the outline of the case; the operation was performed with skill and expedition; the after-treatment of the patient was safe and judicious; but a very important point, which appears to have been overlooked in the history, although we must suppose that it shared the attention of the operator, is the neglecting to state accurately the dimensions of the pelvis. It is true that Dr. S. speaks of not being able to introduce two fingers between the rami ischiorum, but this is a very indefinite mode of measurement; he should have employed the forceps of Baudelocque, an instrument that no accoucheur ought to be without, and, if he did employ it, he ought to have stated exactly the different diameters of the pelvis.

Dr. Meyer's case is also very interesting, the principal points are the following. The patient, a woman 33 years of age, the mother of six children, had, in each labour, no difficulties or impediments to the progress of the parturition, with the exception of the last. During the pregnancy with the sixth child, the patient, who had been before an active and strong little woman, became, through the pressure of domestic misfortunes, very much disordered, so that her health suffered very much, and she became very much weakened and relaxed. The sixth labour was tedious, and, according to the account of the midwife who attended her, the head appeared to be larger than in the former labours. Delivery was effected by the natural efforts, but the patient felt, at the time, as if something had burst in the pelvis, which was immediately followed by a sudden and severe pain. This pain had its seat in the symphysis pubis; after a day or two it diminished, and then attacked the sacro-iliac symphysis, especially on the right side; it extended itself, also, to the left joint, and was so violent that the patient could not find a convenient or easy situation to lie in. The pain gradually subsided, and, whilst remaining in bed, the patient was easy, but, when, after her confinement, she attempted to get up, every motion of the body produced great pain in the pelvis, and it always felt, as she expressed it, as if two hard things were rubbing together, attended with a grating noise audible to the bystanders. From this history, it is very probable that an absolute separation of the ossa pubis, at the symphysis, took place during the delivery; such cases have been before noticed, several such have been described by MICHELL in one of his Commentaries, who not only observed such cases himself, but refers to others who had, before him, seen the same. "Fuerunt enim celebres viri," says Michell,* "qui hunc ossium pelvis secessum in ipso partu viderunt, vel *per strepitum* perceperunt, vel claudicationem post partum observârunt, ut inter alios Veslingius, Soumain, et Dr. Bieker." For two years and a half, the interval between the last confinement and the present, the patient was able only to move about with crutches, and, during the latter part of the present pregnancy she could only move herself about very slowly, and with a great deal of pain, between the table and chairs. If she happened to bend herself forward, it was with the greatest difficulty the upright position was recovered, and she was frequently obliged to call her

* De Synchondrotomia Pubis Commentarius, Amstel, 1783, p. 82.

husband to her assistance. When the patient stood upright, the right foot was full an inch shorter than the left. By a careful examination of the pelvis, it was found that the rami of the ischium and pubes were very much pressed together, the space between them measured only an inch and a half, one finger could be pressed through, but the attempt to introduce two, occasioned great pain. The whole pelvis appeared to be completely knit together, the right os pubis was particularly curved inwards. The motions of the pelvis, on attempting to bend the body or extend it, gave great pain, not only in the symphysis pubis, but also in the sacro-iliac symphysis and the hip-joints; and, under this state of affairs, it was deemed by Dr. Meyer and his colleagues, necessary to deliver the woman by the abdominal section. The patient was so convinced of the great change which her figure had undergone, by what she called the rheumatic gout, that she repeatedly expressed her conviction of the impossibility of being delivered in the natural manner.

*Operation.* On the 15th of March, early in the morning, the labour pains commenced; these continued violent and frequent throughout that day and a great part of the night; the os uteri could scarcely be felt at the upper aperture of the pelvis, and descended no farther during the morning of the 16th. On the 16th, the operation was performed in the linea alba. The incision was commenced two inches below the umbilicus, and extended to within two inches of the symphysis; the upper part of the incision was afterwards enlarged to within an inch of the navel, so that the whole length was six inches. The linea alba was afterwards divided in the same manner, when the bluish-coloured uterus came into view. An opening was made in the upper part of the uterus, the finger was introduced for the purpose of carrying the bistoury on it, to enlarge the wound, when it was discovered that the placenta was attached to its fore-part, and that the incision would, if continued perpendicularly, pass through two-thirds of its circumference. No choice remained; the bistoury was carried rapidly through its structure, and the uterus divided to near the bottom of the external wound. The placenta was of a firm, but of a natural, consistence; and, although the vessels traversing it appeared frightfully large, the bleeding which followed their division was very little and unimportant. Perhaps this was, in some measure, owing to the child being dead, a circumstance before prognosticated by Dr. Meyer, from the woman's having felt a sudden shivering on the first day of the labour. The child was immediately laid hold of, and, without any difficulty, taken out; it showed not the least mark of life. The navel-string was instantly cleared and divided, the hand passed into the uterus, and the two severed portions of the placenta, without any difficulty, separated. Most interesting was it to observe, (says Dr. M.) at this moment, the sudden contraction of the uterus, which quickly diminished to the size of a large goose's egg; the edges of the wounded uterus, however, did not approximate, and I thought I could do no better than follow the advice given by Wigand, and press the uterus a little together with the hand and push it gently deeper in the pelvis. The finger was passed into the uterus, and, through its partially dilated orifice, into the vagina, in order to be assured that no impediment recurred to the passage of the lochia. The blood, which had escaped with some of the liquor amnii, into the abdomen, was carefully absorbed with a sponge. The intestines which protruded, as happens in most cases at the sides of the wound, however carefully the assistants may make pressure, were returned, and the edges of the wound secured with sutures and adhesive plaster; an opening, or a lightly dressed part of the wound, was left about two inches above the pubis to allow of the escape of the fluid from the abdomen and the wound. During the operation, the patient complained very little of pain, and assured the operator and her friends afterwards, that, of her severe labours, this had been the least painful. The operation, from the commencing to the finishing the dressing and putting the patient into bed, occupied twenty-eight minutes. The night was passed well; she had fallen into a short sleep, and had taken afterwards a little gruel with appetite. On the evening of the day following the operation, (the 16th) the patient vomited twice, and complained of a pain in the lower part of the wound, over which the dressings were lightly applied; this was carefully examined, and a portion of omentum that had been pushed forward was returned. At ten the same evening, a little demulcent and opiate mixture was prescribed to quiet a cough, which the patient had been teazed with for several days. She passed the night tranquilly, and slept several hours. The bowels were properly attended to, the skin kept moist, and the general state of the patient was most favourable. On the 19th, the lochia came away through the vagina, and the lower part of the abdominal wound. The condition of the wound continued to be every thing that could be wished; the patient proceeded without fever or abdominal inflammation to a fortunate recovery.

On the 20th of April, the whole wound had cicatrized, with the exception of a minute point at the lower extremity, through which a little matter was discharged. The patient was, with a little assistance, able to move about the chamber without complaining of pain. On the 1st of May, the wound was completely healed, and the patient was in every respect well. Three months after the operation, the menses made their appearance, and that without any pain or inconvenience. We find, by the after history of the case, that this woman visited Dr. Meyer in the month of April of the succeeding year, and that she was then again pregnant; she was informed it would be necessary to repeat the operation, at which intelligence, instead of being dejected or alarmed, she appeared rather pleased, and expressed her hope, that he would perform the operation with the same skill as the first. The cicatrix appeared very firm, but was very much stretched by the distended uterus, so that it was feared that it might give way from the force of the accumulating pressure. On the morning of the 4th of June, Dr. Meyer was called to the woman, as the labour had commenced, but before he could reach the house the woman suddenly died. On examination of the body, it was discovered that the uterus was ruptured throughout the whole length of the former incision, and the edges of the ruptured part were not thicker than common paper. The fœtus, with the membranes entire, lay quite free in the abdomen, in which was also a quantity of coagulated blood. From the appearance of the cicatrix of the linea alba seen from the inside, it was considered that it would have given way during the progress of the labour, as it appeared exceedingly thin, and had already in some places begun to separate. The examination of the pelvis also confirmed the prognosis which had been before given of the separation of the bones of the pelvis; the symphysis pubis was completely separated; not a trace of cartilage remained, even the ligaments were very much altered in texture, and very much weakened, so that by a short maceration, all the bones of the pelvis separated readily from each other.

In the history of these cases, two important facts are elicited, which we have also had an opportunity to observe, and which tend to diminish the danger of the operation; namely, the little bleeding, and the little pain occasioned by its performance. The attestations of many operators bear upon these points. In Professor Mende's last Journal, are two cases of this operation recorded; the one terminated fatally in thirty days, the other in sixty-seven hours; but in both, Dr. BUSCH of Morburg, the operator, remarks "both of these patients complained not so much of the operation itself as of the pain caused by introducing the sutures through the integuments: the hæmorrhage was also slight." Drs. Meyer and Schenk have repeated this observation, which will be seen by a reference to the cases. Mr. WOOD of Manchester, who performed the operation of Lauveijat, says, that there was, in his case, scarcely any effusion of blood either from the external wound, or from that of the uterus, though the latter was made directly upon the placenta.

Dr. Schenk's case shews that the operation does not destroy the functional power of the uterus, nor hinder the process of conception; the woman arrived at the full time of gestation, and would have readily submitted to a second operation. The possibility of a second Cæsarian operation on the same person has been questioned by many; the cases to be found in many publications are too well authenticated to admit of a doubt. In the "Russian Repertorium of the Natural Sciences and the Healing Art," published at Riga, by CRICHTON, REHMANN and BURDACH, is a case recorded, in which the operation was twice performed. The history of a case in which this operation was performed *three* times, is given by the late OSIANDER, in Vol. ii. of the Commentations of the Royal Society of Sciences of Gottingen, of 1813.* In the third volume of SIEBOLD'S *Journal fur Geburtshulfe*, is a case in which the operation was twice performed on the same person.† A French physician, LOUNIUS, is said to have performed this operation *seven* times on the same woman, and that the children so delivered all lived. The Cæsarian operation, formidable as it is, is by no means so fatal as most of our countrymen suppose, and it is very probable that the boldness which the continental surgeons have of late shown, will inspire the former with more confidence than they have hitherto possessed, and induce them to perform it earlier in the duration of the labour than formerly has happened. It must be understood, however, that we would recommend the operation only where the most imperative necessity called for it, and that men must be destitute of every moral principle and common honesty, who would rashly undertake it without having first most scrupulously examined the grounds on which such a necessity rests.

---

* Fr. B. Osiander novam methodum instituendam vivente fœmina ventris gravidi incisionem ab ipso inventam et bis péractam adjectam observationibus huc facientibus prælectione exposuit. Com. Soc. Reg. Scient. Gött. This novam methodum, ab ipso inventam, which Osiander tried to establish, has been very justly considered too trifling a deviation from the ordinary linea alba incision to merit any distinction in the list of operations.

† In a recent number of this work was published a circular letter of Dr. DAVIS, which he addressed to several of the principal accoucheurs of Germany, to obtain the results of their experience on certain points, which Dr. Davis wished to establish in his late work. Dr. Davis's questions were not shaped according to the liking of Professor Siebold, and although addressed to him as a private practitioner, he immediately clapped them into his Journal, accompanied with remarks by no means honourable to either party. This exposure of a private correspondence, has made the tour of Germany, so that now scarcely a medical periodical comes out, in which one does not find "*Answers to the Herr, Dr. Davis's proposed obstetrical questions.*" A breach of good breeding, which has procured for the Editor, the censure of most of his countrymen; a censure in which we join.

